# Trapped in the Trabeculae: A Rare Case of Noncompaction Cardiomyopathy and Large Left Ventricular Thrombus

**DOI:** 10.7759/cureus.106359

**Published:** 2026-04-03

**Authors:** John Bajouka, Christopher Matti, Ghaid Touza

**Affiliations:** 1 Internal Medicine, Henry Ford Health System, Southfield, USA; 2 Medicine, Wayne State University School of Medicine, Detroit, USA

**Keywords:** cardiac imaging - mri, cardiomyopathy, direct oral anticoagulation, left ventricular noncompaction, transthoracic echocardiography (tte), ventricular thrombus

## Abstract

Left ventricular noncompaction (LVNC) is a rare primary cardiomyopathy characterized by a failure of myocardial morphogenesis, resulting in a prominent, characteristic "sponge-like" trabeculated endocardial layer. Clinical presentation is classical for heart failure, ventricular arrhythmias, and systemic thromboembolism, which defines its severity. Here, we describe a 34-year-old male with no prior medical history who presented with progressive dyspnea and volume overload. On initial evaluation, severe biventricular failure, stage 3b chronic kidney disease (type 2 cardiorenal syndrome), and significant hemodynamic impairment were noted. Subsequently, contrast-enhanced transthoracic echocardiography (TTE) was consistent with the diagnosis of LVNC and identified multiple mural thrombi, including a 2 cm mobile apical thrombus. Coronary angiography revealed normal coronary anatomy. Following these findings, the patient was stabilized with intravenous diuretics and initiated on guideline-directed medical therapy (GDMT). Due to the high risk of sudden cardiac death and persistent low ejection fraction, he was discharged with a wearable cardioverter defibrillator and referred for advanced heart failure and transplant evaluation. Shifting focus to the underlying pathophysiology, thrombus formation in LVNC is unique. It goes beyond global systolic dysfunction; the deep intertrabecular recesses create stasis that predisposes to fibrin deposition regardless of wall motion. This thrombogenicity necessitates a nuanced approach to anticoagulation. In summary, LVNC should be a primary consideration in young patients presenting with new-onset heart failure. This case underscores the necessity of imaging to identify obscured thrombi and highlights the evolving role of direct oral anticoagulants (DOACs) in managing the specific mechanical risks inherent to noncompacted myocardium.

## Introduction

Left ventricular noncompaction (LVNC) is a distinct primary cardiomyopathy resulting from the premature arrest of myocardial morphogenesis during the first trimester of embryonic development. During this process, the primitive meshwork of the embryonic myocardium fails to undergo normal compaction into a dense, solid muscle layer, instead retaining a prominent characteristic "sponge-like" architecture [1]. This structural anomaly is characterized by a thin, compacted epicardial layer and an extensively trabeculated endocardial layer with deep intertrabecular recesses that communicate directly with the ventricular cavity. Primarily localized at the apex and midventricular lateral walls [2], LVNC presents a significant clinical challenge due to its association with progressive heart failure, life-threatening ventricular arrhythmias, and systemic thromboembolism [3].

Diagnosis has evolved from purely morphological assessment to a multimodality approach, primarily utilizing standardized echocardiographic criteria, such as the Jenni criteria, which evaluate the noncompacted to compacted (NC/C) ratio at end-systole [4]. Cardiac magnetic resonance (CMR) imaging is also used to aid in the diagnosis of LVNC. CMR remains the gold standard for tissue characterization, employing the Petersen criteria to measure the NC/C ratio at end-diastole greater than 2.3 and to identify late gadolinium enhancement (LGE) to risk-stratify patients for sudden cardiac death. However, there are diagnostic limitations, with high sensitivity but moderate specificity when applied in isolation [5]. Despite these standardized metrics, the heterogeneous nature of LVNC necessitates a high index of suspicion, as the mechanical environment of the recesses predisposes patients to intracavitary thrombi, particularly in the setting of an ejection fraction (EF) of less than 40% [1].

Current clinical challenges include optimizing the timing of anticoagulation and selecting appropriate agents. The aim of this article is to present a case of LVNC in a young adult presenting with heart failure and an apical thrombus. By detailing the imaging approach and subsequent therapeutic strategy, this report highlights the critical importance of early risk stratification and the evolving role of direct oral anticoagulants (DOACs) in managing LVNC-associated thrombus.

This case report has been prepared in accordance with the CARE (CAse REport) guidelines [6]. The authors confirm that all relevant components of the CARE checklist have been addressed to ensure completeness and transparency of reporting.

## Case presentation

A 34-year-old male with no prior medical history presented with two weeks of progressive dyspnea on exertion, orthopnea, and bilateral lower extremity edema. The patient denied anginal symptoms. Vital signs revealed tachycardia at 106 beats per minute, though he remained normotensive. Physical examination was significant for jugular venous distention, mild bibasilar rales, a grade 3/6 holosystolic murmur at the apex, and 3+ bilateral lower extremity pitting edema. Initial laboratory evaluation was significant for a high sensitivity troponin T of 753 ng/L, proBNP of 3,626 pg/mL, and creatinine of 2.0 mg/dL.

**Figure 1:**
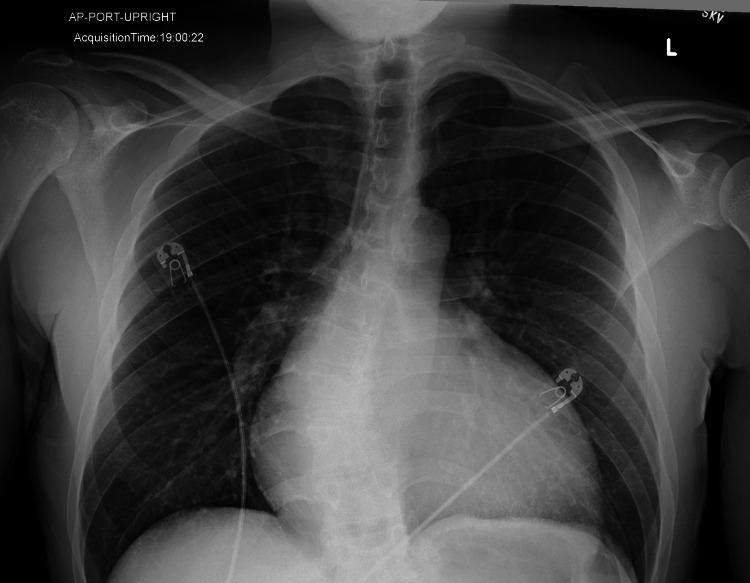
Chest X-ray upon admission Mild interstitial prominence in the lungs related to mild pulmonary vascular congestion. An enlarged cardiac silhouette is noted, consistent with cardiomegaly.

Upon admission, an electrocardiogram showed sinus tachycardia with T-wave inversions in the inferior and lateral leads. A chest X-ray (CXR) was performed, which showed pulmonary vascular congestion (Figure 1). While myocarditis was initially considered due to the elevated troponin levels, this was deemed less likely in the setting of concurrent chronic kidney disease and acute heart failure.

**Figure 2:**
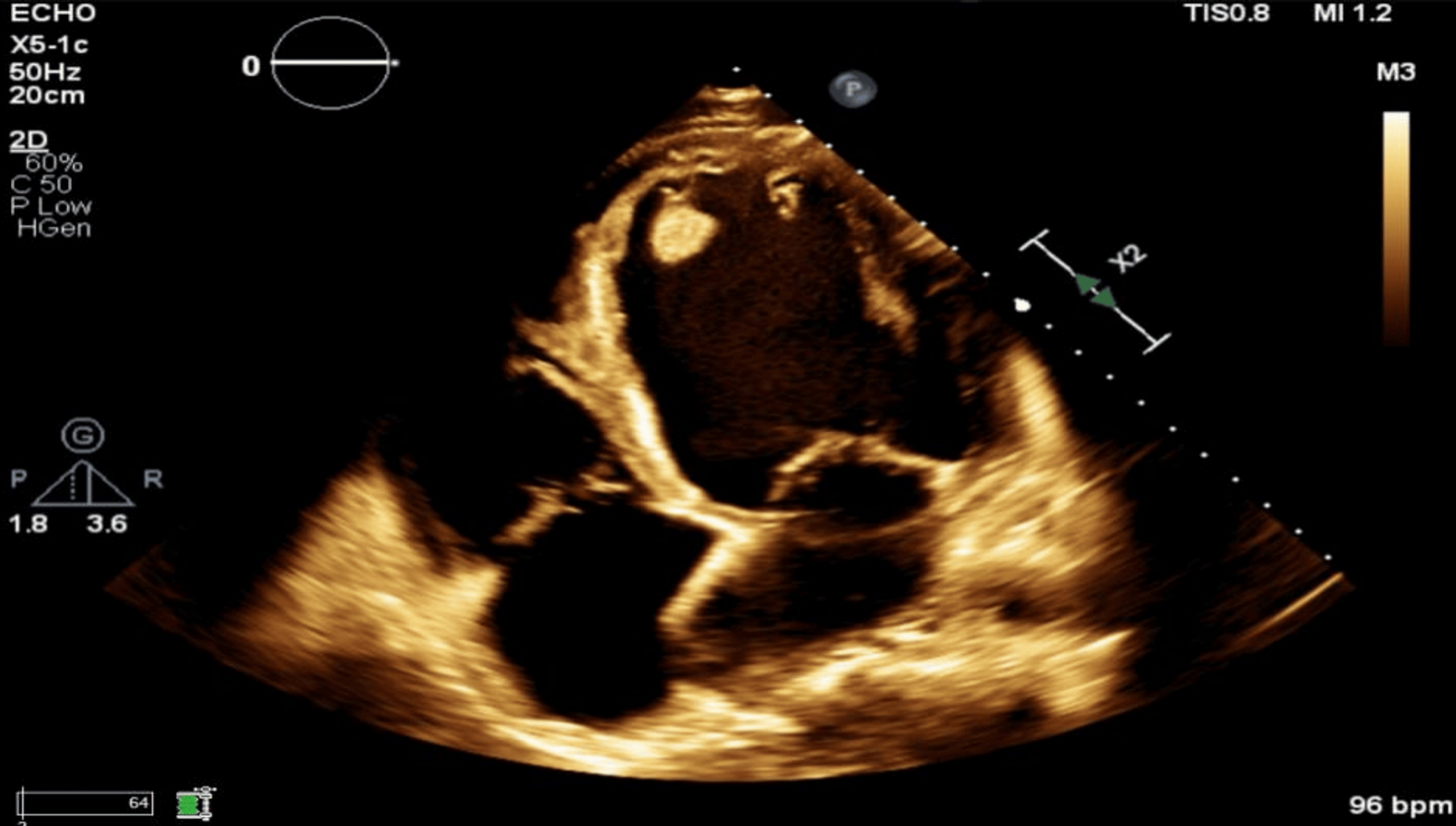
Transthoracic echocardiography, four-chamber view Transthoracic echocardiography in a four-chamber view showing a severely dilated left ventricle. A prominent apical thrombus is noted.

A transthoracic echocardiography (TTE) was obtained and revealed a severely dilated left ventricle with an EF of 10-15%, global hypokinesis, and severe concentric mitral regurgitation. Severely reduced right ventricular (RV) function with a RV systolic pressure of 31 mmHg and tricuspid regurgitation peak velocity of 0.8 m per second was also noted (Figure 2).

**Figure 3:**
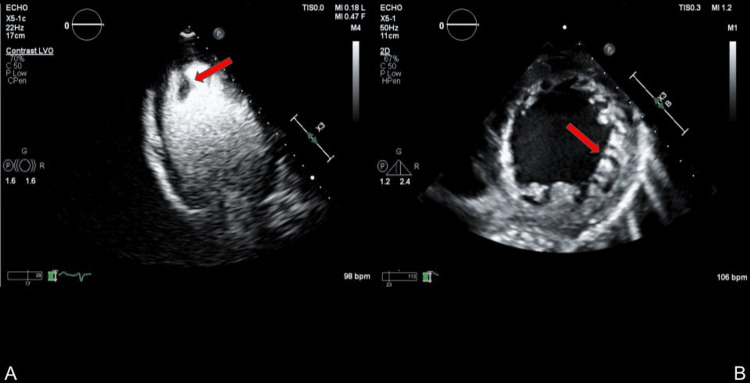
Transthoracic echocardiogram with contrast (A) A 2 cm mobile thrombus at the apex of the left ventricle is visualized. (B) A parasternal short axis view reveals prominent apical trabeculations and deep recesses, consistent with a diagnosis of left ventricular noncompaction (LVNC).

Contrast was administered during the TTE. Prominent apical trabeculations and deep recesses were visualized, consistent with a diagnosis of LVNC. As a limitation, Jenni's echocardiographic criteria were not formally applied. A 2 cm mobile thrombus at the apex was noted (Figure 3).

**Figure 4:**
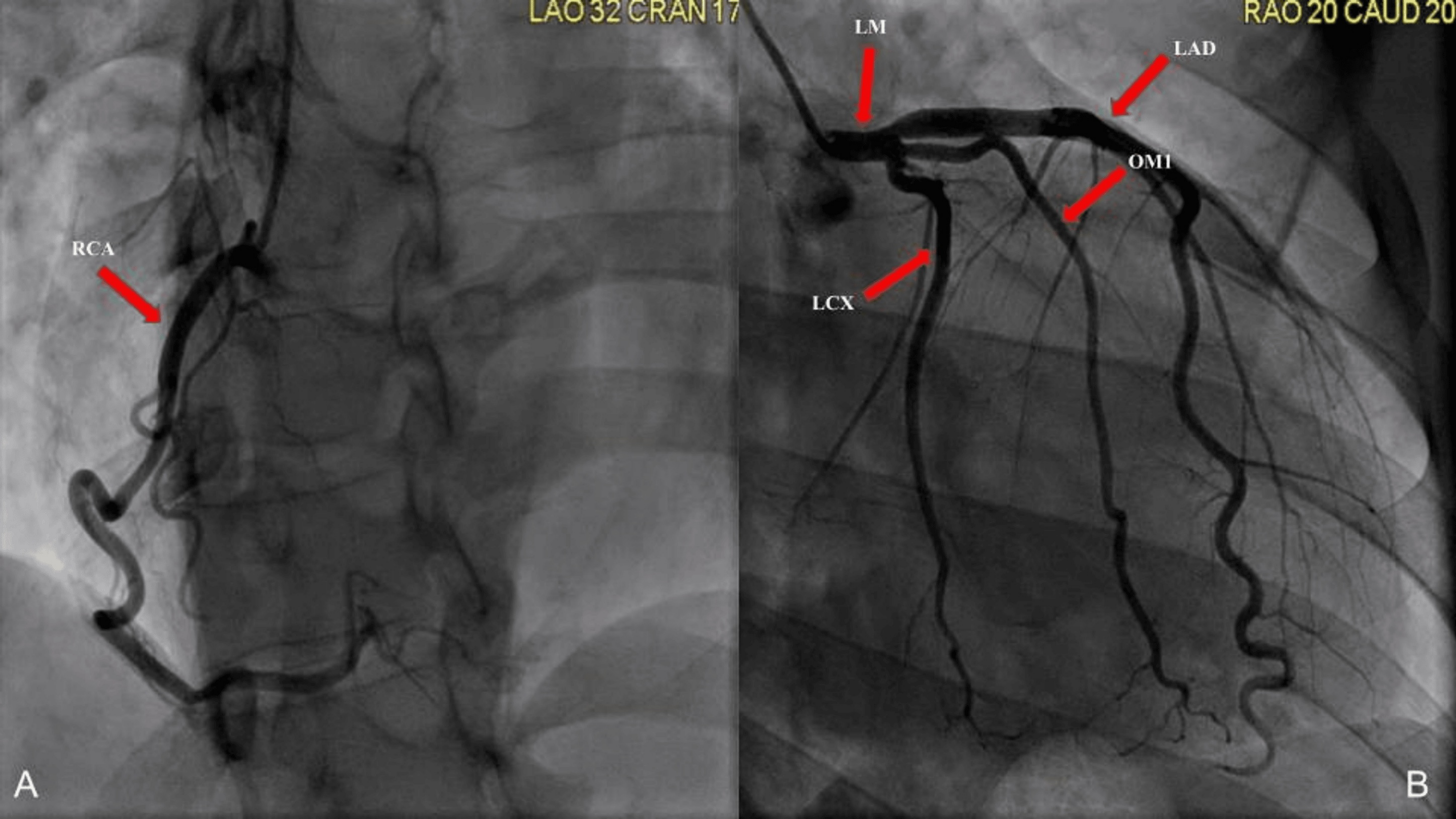
Selective coronary angiography (A) Right coronary artery with no evidence of coronary artery disease. (B) Left coronary system with major branches showing normal anatomy and no signs of arterial disease. RCA = Right coronary artery, LM = Left main coronary artery, LAD = Left anterior descending artery, LCX = Left circumflex artery, and OM1 = 1st obtuse marginal artery

The patient was treated with intravenous furosemide and initiated on a heparin drip for acute anticoagulation. Left-sided cardiac catheterization was performed, which confirmed normal coronary anatomy with no obstructive disease noted (Figure 4).

A right-sided cardiac catheterization was also performed, confirming a low cardiac output of 2.99 L/min (Table 1) and evidence of pulmonary hypertension (Table 2).

**Table 1:** Right heart catheterization hemodynamic profile with oxygen saturation and cardiac output This table summarizes invasive hemodynamic measurements obtained during right heart catheterization, including intracardiac pressures, pulmonary artery oxygen saturation, and cardiac output/index calculated using the Fick method. Measured values are presented alongside standard reference ranges to facilitate interpretation of abnormalities in preload, afterload, pulmonary vascular resistance, and overall cardiac performance. RA = Right atrium, RV = Right ventricle, PA = Pulmonary artery, PCWP = Pulmonary capillary wedge pressure, LV = Left ventricle, EDP = End-diastolic pressure, O₂ Sat = Oxygen saturation, CO = Cardiac output, CI = Cardiac index

Site	Systolic (mmHg)	Diastolic (mmHg)	Mean/EDP (mmHg)	O2 Sat (%)	CO/CI	Reference Range
Right Atrium	15	12	13.5	-	-	Mean: 2-6 mmHg
Right Ventricle	35	3	4	-	-	Sys: 15-30, Dia: 2-8
Pulmonary Artery	37	14	26	53	-	Sys: 15-30, Dia: 4-12, Mean: 10-20
PCWP	18	19	16	-	-	Mean: 6-12 mmHg
Left Ventricle	82	4	11	-	-	Sys: 100-140, EDP: 3-12
Aorta	110	88	96	92	-	Sys: 100-140, Dia: 60-90
Cardiac Output/Index	-	-	-	-	2.99/1.56	CO: 4-8 L/min, CI: 2.5-4.0

**Table 2:** Calculated vascular resistance indices derived from right heart catheterization This table presents calculated hemodynamic parameters derived from right heart catheterization data, including oxygen utilization metrics and indices of pulmonary/systemic vascular resistance. Values were obtained using the Fick principle and standard hemodynamic formulas. Reported measurements are compared with established reference ranges to aid in assessing cardiac output, tissue oxygen extraction, and the presence of pulmonary or systemic vascular dysfunction. A-V O₂ Diff = Arteriovenous oxygen difference; VO₂ (O₂ Consumption) = Whole-body oxygen consumption; PVR = Pulmonary vascular resistance (resistance within the pulmonary circulation); SVR = Systemic vascular resistance (resistance within the systemic circulation); TPR = Total pulmonary resistance (total resistance across the pulmonary vasculature); TSR = Total systemic resistance (total resistance across the systemic vasculature): TPR/TSR Ratio = Ratio of pulmonary to systemic vascular resistance; dyn·s/cm⁵ = Standard unit of vascular resistance

Parameter	Value	Units	Reference Range
A-V O_2_ Diff	87.1	mL/L	40-60
O_2_ Consumption	260.9	mL/min	200-250
Pulmonary Artery Resistance	267.6	dyn·s/cm^5^	20-130
Systemic Vascular Resistance	2541.8	dyn·s/cm^5^	800-1200
Total Pulmonary Resistance	695.7	dyn·s/cm^5^	< 250
Total Systemic Resistance	2568.6	dyn·s/cm^5^	800-1200
TPR/TSR Ratio	0.27	-	< 0.1-0.15

Following clinical stabilization, the patient was initiated on guideline-directed medical therapy (GDMT), including sacubitril/valsartan, carvedilol, spironolactone, and empagliflozin. In light of the patient's renal impairment (estimated glomerular filtration rate (eGFR) ~40 mL/min/m²), sacubitril/valsartan was initiated at a low dose (24/26 mg twice daily) to ensure tolerability. He was transitioned from heparin to 5 mg of apixaban twice daily for long-term anticoagulation. Given the persistent low EF and the high risk for sudden cardiac death, he was discharged with a wearable cardioverter defibrillator and referred to electrophysiology and advanced heart failure specialists for further evaluation for an implantable cardioverter defibrillator (ICD) and cardiac transplantation.

## Discussion

The pathophysiology of thrombus formation in LVNC represents a unique clinical paradigm distinct from standard dilated or ischemic cardiomyopathies. While traditional thrombi are typically the result of endocardial injury or global blood stasis secondary to a low EF, in LVNC, the risk is fundamentally structural and independent of systolic function [7]. The characteristic "sponge-like" morphology of the non-compacted layer creates deep intertrabecular recesses that function as isolated compartments. Blood flow becomes sequestered from the high-velocity laminar flow within the central ventricular cavity, creating a localized environment of stasis [8].

Furthermore, the anatomical complexity of the trabeculated endocardium exponentially increases the surface area available for clot attachment. Due to the mechanical web-like ventricular wall in LVNC, thrombus formation becomes more stable and potentially more difficult to visualize on standard 2D imaging without contrast [8]. In this patient, the 2 cm mobile apical thrombus is likely multifactorial, related to non-compacted recesses combined with stasis due to severe global hypokinesis. This structural predisposition necessitates a more aggressive and nuanced approach to anticoagulation, as the risk of systemic embolization is driven by the endocardial pathology rather than solely by contractile failure [9].

Although, in this case, the patient had a visualized thrombus and anticoagulant decision-making was straightforward, the clinical decision to initiate anticoagulation in patients with LVNC often resides in the physician's judgment when a thrombus is not immediately visualized [7,10]. While older studies suggested anticoagulating all LVNC patients, a recent study emphasizes a risk-stratified approach. Current evidence suggests that the risk of thromboembolism is greater when systolic function is impaired, with a strong clinical lean toward prophylaxis in patients with a left ventricular EF (LVEF) < 40% [11]. Conversely, in isolated LVNC with preserved EF and no history of atrial fibrillation, morphological hypertrabeculation alone does not appear to significantly increase the risk of embolization [10,11].

While vitamin K antagonists (VKAs) such as warfarin have historically been the gold standard for mobile thrombi due to extensive long-term data, the evolving evidence through 2026 increasingly favors DOACs [7]. The REsolution of LEft VENTricular thrombus (RELEVENT) trial [12] is an ongoing randomized trial comparing DOACs vs. VKAs for LV thrombus resolution, reflecting clinical equipoise between these agents. A large retrospective cohort study using the TriNetX database [13] compared DOACs and VKAs for LV thrombus over 90 days, reporting comparable rates of stroke (RR: 0.859), major bleeding (RR: 0.902), and systemic embolism, with a favorable safety profile for DOACs. In the setting of stage 3b chronic kidney disease with a GFR of approximately 40, 5 mg twice daily of apixaban offers a more predictable pharmacological profile and may mitigate the risk of bleeding complications compared to VKAs [7]. However, the decision remains a fine balance between the mechanical risk of the trabeculae and the patient's renal clearance, underscoring the need for a multidisciplinary strategy involving advanced heart failure and electrophysiology specialists to manage the concurrent risks of sudden cardiac death and systemic thromboembolism.

CMR imaging is critical for risk stratification in LVNC, providing prognostic insights beyond morphology. Given institutional factors, including unavailability, a CMR was never performed. LGE independently predicts adverse outcomes, including heart failure hospitalizations, arrhythmias, and cardiac death. It is noted that non-compaction degree may not correlate with clinical decline, whereas LGE-identified fibrosis and reduced global LVEF are primary indicators of poor prognosis. Thus, CMR-based tissue characterization identifies high-risk patients for targeted surveillance or early therapeutic interventions, such as ICD consideration [14].

## Conclusions

LVNC remains a complex and high-risk clinical entity, particularly in young patients presenting with new-onset heart failure and reduced EF. This case highlights the critical importance of a multimodality imaging approach; while contrast-enhanced echocardiography was diagnostic in this case, CMR may further guide prognosis and risk stratification in clinically stable patients. The management of LVNC-associated thrombus presents a unique challenge, as the risk of thrombus formation is driven not only by global systolic dysfunction but also by stasis inherent to the non-compacted myocardium.

Ultimately, improving outcomes in LVNC requires early recognition of heart failure and thromboembolism. A multidisciplinary approach to aggressive guideline-directed medical therapy is essential. Early referral to advanced heart failure and electrophysiology specialists is critical in managing the long-term risks of sudden cardiac death and potential progression to cardiac transplantation.
